# Formation of phenylacetic acid and phenylpropionic acid under different overload conditions during mesophilic and thermophilic anaerobic digestion

**DOI:** 10.1186/s13068-019-1370-6

**Published:** 2019-02-10

**Authors:** Andreas Otto Wagner, Eva Maria Prem, Rudolf Markt, Rüdiger Kaufmann, Paul Illmer

**Affiliations:** 1 Department of Microbiology, Universität Innsbruck, Technikerstraße 25d, 6020 Innsbruck, Austria; 2 Department of Ecology, Universität Innsbruck, Sternwartestr. 15/Technikerstraße 25/5, 6020 Innsbruck, Austria

**Keywords:** Anaerobic digestion, Phenylacetic acid, Phenylpropionic acid, PAA, PPA, Biogas, Methane

## Abstract

**Background:**

Substrate spectra for anaerobic digestion have been broadened in the past decade, inter alia, due to the application of different pretreatment strategies and now include materials rich in lignocellulose, protein, and/or fat. The application of these substrates, however, also entails risks regarding the formation of undesired by-products, among which phenolic compounds are known to accumulate under unfavorable digestion conditions.

**Methods:**

Different states of overload were simulated in batch experiments while reviewing the generation of phenyl acids out of different lab-use substrates in order to evaluate the impact on biogas and methane production as well as some additional process performance parameters under defined laboratory conditions. Investigations were conducted under both mesophilic and thermophilic conditions.

**Results:**

It could be shown that the tested input materials led to the formation of phenyl acids in a substrate-dependent manner with the formation itself being less temperature driven. Once formed, the formation of phenyl acids turned out to be a reversible process.

**Conclusions:**

Although a mandatory negative impact of phenyl acids per se on the anaerobic digestion process in general and the methanogenesis process in particular could not be proven, phenyl acids, however, seem to play an important role in the microbial response to overloaded biogas systems.

**Electronic supplementary material:**

The online version of this article (10.1186/s13068-019-1370-6) contains supplementary material, which is available to authorized users.

## Introduction

In the past decade, anaerobic digestion has gained increasing importance in both treating different (waste-)substrates and generating energy from biomass in general. Hence, various improvements were suggested [1] and substrates spectra have been extended including (pretreated) lignocellulosic biomass [2] and protein-rich substrates such as industrial, kitchen, and food wastes [3]. However, the application of these substrates also poses risks regarding the formation of undesired by-products. Among these, phenolic compounds are known to accumulate under unfavorable digestion conditions and to exert a possible negative effect on the anaerobic digestion processes by causing reduced digester performances or even digester failures [4–7].

Aromatic compounds per se are (next to carbohydrates) the second most abundant class of organic compounds in nature [8], which are (dependent on the availability of oxygen) microbiologically degraded by two major strategies. While the aerobic catabolism has been studied for several decades [9, 10], the anaerobic degradation of aromatics is a more recently discovered microbial capacity that still requires a deeper understanding despite the fact that microbial metabolism in the absence of oxygen is the most ancient of all life processes [11–13]. The mineralization of aromatic compounds by facultative or obligate anaerobic bacteria (and some archaea) can be coupled to anaerobic respiration with a variety of electron acceptors, e.g., nitrate, sulfate, iron(III), manganese(II), and selenate, with each one conserving different yields of energy [11]. The benzoyl-CoA pathway appears to be the most important one in the degradation of aromatic substances as a broad variety of compounds enter this path, including phenol, various hydroxybenzoates, phenylacetate, aniline, certain cresols and even the pure hydrocarbon toluene [14–18]. Anaerobic degradation of aromatic compounds can be found in sulfate and iron reducing, as well as fermentative bacteria. To keep fermentation product concentrations low, a syntrophic cooperation of an aromatic fermenting and a methanogenic or sulfate reducing organism is essential [10, 19].

The inhibitory or toxic effect of aromatic compounds on the anaerobic digestion process, however, has to be discussed in view of factors like operation mode, microbial community composition, and various physico-chemical parameters [6, 20]. The degradation efficiency and pathway of different aromatic compounds were shown to be influenced by the microbial community structure and the operational temperature [21–28]. Data on the anaerobic degradability are available for various aromatic compounds including phenols, chloro-, nitro-, and bisphenols, phthalates, and endocrine disrupting compounds [28–33], whereas the phenyl acids phenylacetate (PAA) and phenylpropionate (PPA), which can be found in anaerobic digestion plants treating kitchen [4], olive oil mill [34], or citrus processing [35] residues, but also in swine manure [36], have received little scientific attention. PAA and PPA were also identified as breakdown products of lignin derivatives or aromatic acids [37–39]. Carbol et al. [6] identified PAA as a major toxic compound during the anaerobic digestion process and found substrate-dependent effects on methanogenic activity and archaeal community structure when investigating the effect of PAA pulses, whereas Sierra-Alvarez and Lettinga [40] observed an inhibition of acetoclastic methanogens in granular sludge with PAA but not with PPA. Sabra et al. [41] recorded unstable reactor conditions at PAA concentration up to 0.25 g L^−1^ or inhibitory effects with values above 0.5 g L^−1^. However, PAA was also used as a supplement during anaerobic digestion [42] and a positive effect of PAA [43] but also PPA [44, 45] on the growth of the cellulose degrader *Ruminococcus albus* was in discussion. An organism known to produce phenylacetic acid is *Porphyromonas* (formerly *Bacteroides*) *gingivalis* (from phenylalanine) [46].

The hypothesis of this study was that anaerobic digesters under overload conditions—which occur when the amount of organic matter in a methanogenic habitat exceeds the total microbial capacity to be degraded—can lead to the accumulation of phenyl acids that subsequently impact the overall digestion and/or methanogenesis process. Therefore, the aim of the present study was to (i) simulate different states of overload using different substrates while reviewing the generation of phenyl acids and (ii) to evaluate the impact on biogas and methane production. Investigations were performed under mesophilic and thermophilic conditions, respectively, using inocula derived from large-scale digestion plants applying the respective conditions. The present study mainly deals with the approach to show the formation of phenyl acids from protein-rich substrates and aromatic amino acids and their effect on the anaerobic digestion process in a descriptive manner. A further study describing the dynamics of the microbial community during these experiments is under progress at the time of writing this document.

## Materials and methods

### Experimental setup and design

Serum flasks containing 48 mL carboxymethylcellulose medium (CMC medium, see “Medium” section) as well as different additional substrates in different concentrations were inoculated with 12 mL of diluted sludge (25%) either from a thermophilic or a mesophilic digestion plant. All variations were conducted in three replicates resulting in a total of 39 reactors per incubation temperature. The flasks were incubated at 37 °C and 52 °C, respectively, for 28 days to investigate the formation of the various phenyl acids represented by phenylpropionic acid (PAA), phenylpropionic acid (PPA), and phenylbutyric acid (PBA) under different overload conditions. To allow conclusions on the effect of phenyl acid formation on the entire digestion process, analyses of gas production (overpressure), gas composition (GC analysis), and pH (via indicator strips) as well as various organic acids and alcohols were conducted to assess overall reactor performance.

### Medium

As a basic medium CMC medium (CMCM) was used as it should provide all necessary nutrients to establish a microbial community able to perform the four key digestion phases involving hydrolysis, acido- and acetogenesis, and methanogenesis. CMCM contained per 900 mL a. dest. [47]: 1.0 g NaCl, 0.4 g MgCl_2_ × 6 H_2_O, 0.2 g KH_2_PO_4_, 0.5 g KCl, 0.15 g CaCl_2_ × 2 H_2_O, 0.5 g l-cysteine, 5.0 g sodium carboxymethylcellulose (CMC), 1.0 g yeast extract, and 1 mL resazurin solution (containing 1.15 mg mL^−1^ resazurin). As a buffer system 0.1 M KH_2_PO_4_ (A) and 0.1 M NaOH (B) was used by adding 50 ml A and 45 mL B and bringing it to a final volume of 100 mL. Finally, 1 mL of a filter sterilized vitamin solution (containing per liter: 0.05 g cyanocobalamin, 0.05 g 4-aminobenzoic acid, 0.01 g d-biotin, 0.1 g nicotinic acid, 0.025 g d-pantothenic acid, 0.25 g pyridoxine, 0.18 g thiaminium chloride HCl), 1 mL of a filter sterilized trace mineral solution (containing per liter: 1.5 g FeCl_2_ × 4 H_2_O, 0.07 g ZnCl_2_, 0.1 g MnCl_2_ × 4 H_2_O, 0.19 g CoCl_2_ × 6 H_2_O, 0.002 g CuCl_2_ × 2 H_2_O, 0.024 g NiCl_2_ × 6 H_2_O, 0.036 g Na_2_MoO_4_ × 2 H_2_O, 0.006 g H_3_BO_3_, 10 mL HCl 25%, 0.003 g Na_2_SeO3 × 5 H_2_O, 0.004 g Na_2_WO_4_ × 2 H_2_O, 0.5 g NaOH) and 2 mL sodium sulfide solution (containing 120 g L^−1^ Na_2_S) were added. The pH of the medium was adjusted to pH 7.0. The medium was portioned into 120 mL serum flasks (48 mL each) that were closed using butyl rubber septa, with the headspace being exchanged with N_2_ and CO_2_ (70:30) using an automated gasing machine (GRI, the Netherlands) by applying vacuum and overpressure cycles.

### Substrates

Meat extract and casein as complex protein-rich substrates in final concentrations of 5.0, 20.0, and 50.0 g L^−1^ and the aromatic amino acids phenylalanine, tyrosine, and tryptophan in final concentrations of 1.0 and 10.0 g L^−1^ were used as substrates to simulate different overload conditions. According to supplier information (Carl Roth, Germany) meat extract contained 0.97 g 100 g^−1^phenylalanine, 1.68 g 100 g^−1^ tyrosine, and 0.97 g 100 g^−1^ tryptophan. The addition of amino acid to achieve higher concentrations was not carried out since this would have corresponded, i.e., for phenylalanine to an equivalent of more than 1 kg of meat. The starting C/N ratios spanned from 4 to 12. Medium without substrate addition functioned as a control. According to the applied starting carbon load (measured concentrations in the liquid phase), samples were grouped into control (TC = 4.14–4.42 g carbon L^−1^), as well as low (TC = 4.8–6.0 g carbon L^−1^), medium (TC = 6.1–11.0 g carbon L^−1^), and high load (TC  = 18–22 g carbon L^−1^) (please refer to Table 1).

**Table 1:** Starting total carbon (TC) concentrations (mean ± SD) in the liquid phase and calculated COD (mean ± SD) of flasks containing different substrates at various overload levels

Substrate	Overload	Mesophilic		Thermophilic	
		TC [g L^−1^] (± SD)	COD^a^ (± SD)	TC [g L^−1^] (± SD)	COD^a^ (± SD)
Control		4.4 (0.49)	17.9 (1.22)	4.2 (0.08)	17.8 (1.20)
Tryptophan	Low	4.8 (0.12)	20.1 (0.43)	5.1 (0.03)	22.4 (0.5)
Tryptophan	Medium	7.5 (0.21)	26.8 (1.43)	7.9 (0.23)	28.7 (0.26)
Tyrosine	Low	4.8 (0.07)	20.5 (0.96)	5.1 (0.21)	21.9 (0.76)
Tyrosine	Medium	6.4 (0.32)	28.9 (1.78)	6.1 (0.17)	27.8 (1.4)
Phenylalanine	Low	4.9 (0.26)	21.7 (0.39)	5.1 (0.09)	22.5 (0.7)
Phenylalanine	Medium	9.5 (0.24)	34.1 (1.18)	9.2 (0.30)	35.6 (0.05)
Meat extract	Low	5.8 (0.21)	23.6 (0.61)	5.9 (0.06)	25.6 (0.26)
Meat extract	Medium	10.3 (0.34)	35.5 (1.39)	9.3 (0.73)	35.2 (0.77)
Meat extract	High	21.0 (0.83)	60 (1.19)	18.1 (0.72)	57.5 (0.97)
Casein	Low	5.9 (0.04)	23.5 (0.31)	6.0 (0.19)	24.4 (0.25)
Casein	Medium	10.8 (0.27)	34.1 (0.89)	10.1 (0.35)	36.9 (0.24)
Casein	High	21.5 (0.71)	65.3 (2.57)	20.4 (0.51)	64.2 (0.7)

^a^Calculated from NPOC according to Dubber and Gray [48]

### Inocula

To examine the impact of thermophilic inoculation, digester sludge from the 900,000-L plug-flow anaerobic digestion plant in Roppen/Austria was used, whereas the mesophilic inoculum derived from a co-substrate utilizing waste-treatment plant in Zirl/Austria. To enable liquid handling, the sludge was diluted with oxygen-free distilled water under anaerobic conditions prior to its use as described before [49]. All inocula were pre-incubated for at least 7 days to stabilize the microbial community and to consume potential residual-substrate. For a description of running parameters of the biogas reactor in Roppen as well as detailed chemical, physical, and biological properties of the sludge please refer to previous investigations [50, 51]. For parameters regarding the sludge and the plant in Zirl, please refer to [52]. Basic characteristics of sludge and the digestion plants the inocula were derived from can be found in Table 2.

**Table 2:** Sludge characteristics (undiluted sludge) (mean ± SD) and some basic parameters of the digestions plants the inocula were derived from [50–54]

Parameter	Mesophilic inoculum	Thermophilic inoculum
Reactor capacity (m^3^)	1350	900
Sampling point	Effluent	Reactor outlet
Operation temperature (°C)	39 (0.2)	53 (0.3)
pH	7.36 (0.21)	7.9 (0.44)
Total solids (TS) (g 100 g^−1^ FW)	2.2 (0.04)	26.2 (2.0)
Volatile solids (VS) (g 100 g^−1^ TS)	61.0 (1.89)	
NH_4_-N (mg N kg^−1^)	1385 (128)	3200 (460)
Acetate (g kg^−1^)	< 0.1	1.9 (1.52)
Propionate (g kg^−1^)	< 0.1	1.3 (1.15)
Methanogenic community dominated by	*Methanosaeta* sp.	*Methanothermobacter* sp., *Methanoculleus* sp.

*FW* fresh weight

### Analysis

Sample preparation and analysis of volatile fatty acids, organic acids, phenyl acids, and alcohols (formate, acetate, propionate, iso-butyrate, butyrate, iso-valerate, valerate, lactate, phenylacetic acid, phenylpropionic acid, phenylbutyric acid, methanol, ethanol) via HPLC–UV/VIS or HPLC–RI followed the procedures described in [52]. Concurrently, analyses at 270 nm were conducted to cross-check the presence of PAA, 3-PPA, and 3-PBA, as phenyl acids exhibit strong absorption spectra in this wavelength range due to their aromatic structure and can thus be distinguished from other acids. The parameter “sum of VFA” represents the calculated sum of concentrations of C2–C5 VFAs. Total carbon (TC), non-purgeable organic carbon (NPOC), and total nitrogen (TN), each extracted from the liquid phase, were quantified using a Shimadzu TOC analyzer (Shimadzu, Japan) according to the manufacturer’s protocol after a dilution of at least 1:100. NH_4_Cl and potassium hydrogen phthalate (C_8_H_5_KO_4_) were used as a reference standard. TC and TN were measured in the diluted and NPOC in the diluted and acidified samples (1.5% of 1 M HCl), respectively, according to the manufacturer’s recommendation. For TOC analysis, acidified samples were sparged with hydrocarbon-free air (Messer. Austria) for 10 min. NH_4_-N was measured via HPLC on a Shimadzu Prominence equipped with a fluorescence detector using a flow injection analysis setup (FIA), whereby an HPLC column was replaced with a sample mixing loop. The analysis was established using *ortho*-phthaldialdehyde (OPA) under thiolic-reducing conditions [*N*-acetylcysteine (NAC)], which in the presence of NH_4_ forms a fluorometrically detectable isoindole (ex: 420 nm, em: 500 nm) [55, 56]. As a solvent 5 mM OPA, 5 mM NAC, 5 mM EDTA in 25 mM phosphate buffer (pH 7.6) was used with a flow rate of 0.28 mL min^−1^, an oven temperature of 60 °C, and an injection volume of 5 µL.

### Calculations

Theoretical biogas and methane production was calculated according to VDI 4630 [57] applying a theoretical yield for carbohydrates of 750 mL biogas consisting of 50% CH_4_, for amino acid or protein-rich substrates 800 mL with 60% CH_4_. Concentrations of free ammonia (NH_3_) were calculated according to [58].

Data obtained throughout the study were used to calculate Gibb’s free energies of degradation of aromatic compounds. Using the Nernst equation, values were adjusted to the actually measured concentrations of reaction educts and products. VFA concentrations were taken into account in molar concentrations and CO_2_ and H_2_ as partial pressures in the headspace of reactors. Standard Gibb’s free energies (∆G^0^′) were calculated applying standard free enthalpy of formation (∆G_f_^0^) based on the literature data [59, 60]. ∆G_f_^0^ values for phenylacetate (− 202.4 kJ) and phenylpropionate (− 192 kJ) were derived from [19]; a temperature correction was done according to [59, 61]. For reactants lacking a concentration > 0, the value for the limit of detection divided by 2 was used. PAA and PPA degradation reactions suggested by [19, 62] and ∆G^0’^ values can be found in Table 3.

**Table 3:** Standard Gibbs free energies (∆G^0′^) of different degradation reactions of phenylacetate (C_8_H_7_O_2_^−^) and phenylpropionate (C_9_H_9_O_2_^−^)

No	Reaction^a^	∆G^0′^ [kJ mol^−1^ substrate]	
1	C_8_H_7_O_2_^−^ + 8 H_2_O → 3 CH_3_COO^−^ + 2 CO_2_ + 6 H_2_ + 2 H^+^	+ 123.1	Phenylacetate conversion to acetate, CO_2_ and H_2_ via α-oxidation
2	C_9_H_9_O_2_^−^ + 2 H_2_O → C_8_H_7_O_2_^−^ + CO_2_ + 3 H_2_	+ 73.6	Phenylpropionate conversion to phenylacetate, CO_2_ and H_2_ via α-oxidation
3	C_9_H_9_O_2_^−^ + 10 H_2_O → 3 CH_3_COO^−^ + 3 CO_2_ + 9 H_2_ + 2 H^+^	+ 196.7	Phenylpropionate conversion to acetate, CO_2_ and H_2_ via α-oxidation

^a^Reactions according to [19]

### Statistical analysis

Statistical analysis and graphic processing were performed by using the Software package *Statistica 12* (StatSoft^®^), *SigmaPlot 14* (Systat Software Inc.), and *Rstudio* *version 1.1.453 *(*R version 3.5.1*). If not otherwise indicated results are given as mean ± standard deviation from three replicate samples. Correlations were calculated non-parametrically by *Spearman* *R*_Sp_ using *Statistica 12*. *Kruskal*–*Wallis* ANOVA and *Conover’s* test, including the *Bonferroni* adjustment for p values, was done in * Rstudio* with the *R* package *PMCMR* [63] and *Conover*–*Iman Test of Multiple Comparisons Using Rank Sums by Alexis Dinno*. A significance level of 0.05 (*p* < 0.05) was used to assess differences between treatments.

## Results and discussion

### Mesophilic conditions

#### Reactor performance

Anaerobic digestion of the aromatic amino acids tryptophan, tyrosine, and phenylalanine as well as the complex protein-rich substrates meat extract and casein in different concentrations resulted in successful methane production, although tested under varying overload conditions. While the controls and amino acid containing samples in both tested concentrations (1.0 and 10.0 g L^−1^) yielded similar outputs showing a final methane concentration of approx. 50% after 28 days of incubation, respectively, the addition of complex, protein-rich substrates in low (5.0 g L^−1^) and medium concentrations (20.0 g L^−1^) led to a final methane concentration of up to 60% (± 1.9%) and 68% (± 0.5%), respectively. In contrast, adding 50.0 g L^−1^ protein-rich substrate (high load) yielded a maximum of 37% (± 2.3%) methane in the headspace and, therefore, was lower compared with the control samples, thus clearly reflecting the overload conditions. Hydrogen was detected during the first 4 days in samples containing complex substrates. Consistent with the findings obtained during a previous study using yeast extract as substrate [64], up to 16%(± 1.6%) H_2_ could be detected in meat extract samples (high load), indicating a highly active hydrolytic microbial community (please also refer to Additional file 1). Hydrogen production also occurred in meat extract containing samples in low and medium concentrations, however, in a clearly reduced extent. Regardless of the substrate and initial concentration, hydrogen was used up after 7 days of mesophilic incubation.

**Fig. 1:**
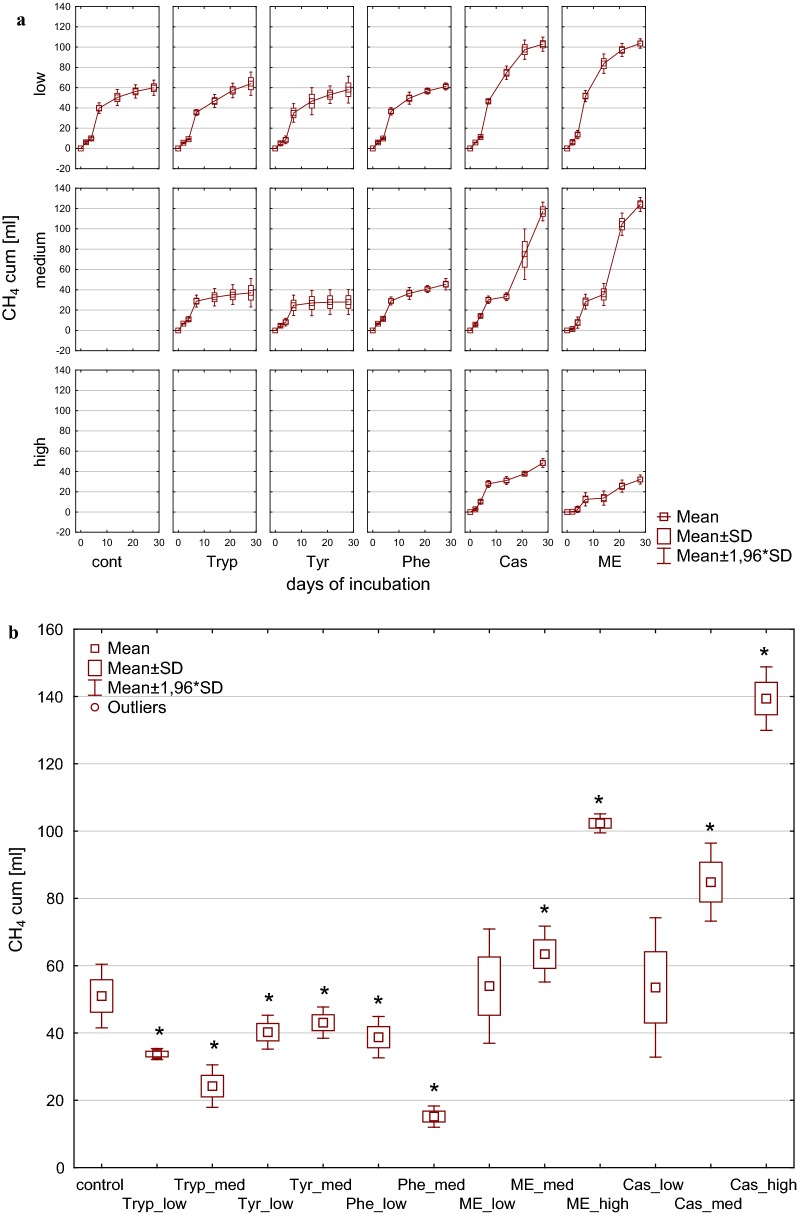
Cumulative methane production during (**a**) and at the end of (**b**) 28 days of mesophilic incubation from reactors reflecting different overload conditions (low, medium, high). *Cont* control, *Tryp* tryptophan, *Tyr* tyrosine, *Phe* phenylalanine, *ME* meat extract, *Cas* casein. *Significantly different from control: conover test. *α* = 0.01. *H*_0_ rejected if *p *≤ *α*/2

**Fig. 2:**
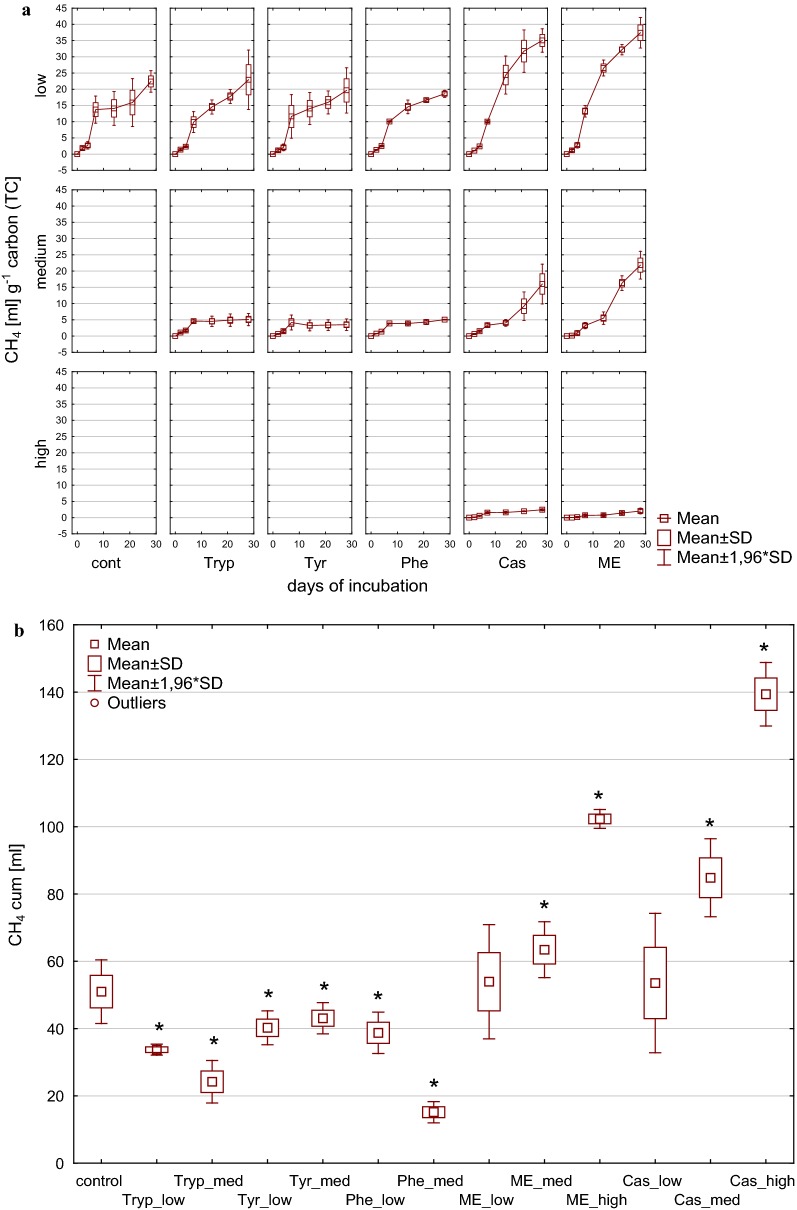
Methane yield per g carbon [mL CH_4_ g^−1^ TC] during (**a**) and at the end of (**b**) 28 days of mesophilic incubation from reactors reflecting different overload conditions (low, medium, high). *Cont* control, *Tryp* tryptophan, *Tyr* tyrosine, *Phe* phenylalanine, *ME* meat extract, *Cas* casein. *Significantly different from control: conover test. *α* = 0.01. *H*_0_ rejected if *p *≤ *α*/2

Cumulative methane yield after 28 days of incubation as depicted in Fig. 1 resulted in significant differences between the tested substrates. Low and medium load conditions from meat extract and casein tended to cause significantly higher overall methane production after 28 days compared with the control, whereas high load impeded overall biogas and methane formation. This effect became even more apparent when calculating the methane yield per carbon unit [mL CH_4_ g^−1^ TC] as shown in Fig. 2. While amino acids (low load) and complex substrates (medium load) resulted in carbon to methane rates similar to those observed in the controls with complex substrates (low load), the methane production per carbon unit was increased, whereas from amino acids (medium load) and complex substrates (high load) a significantly reduced methane production per carbon unit was observed.

**Fig. 3:**
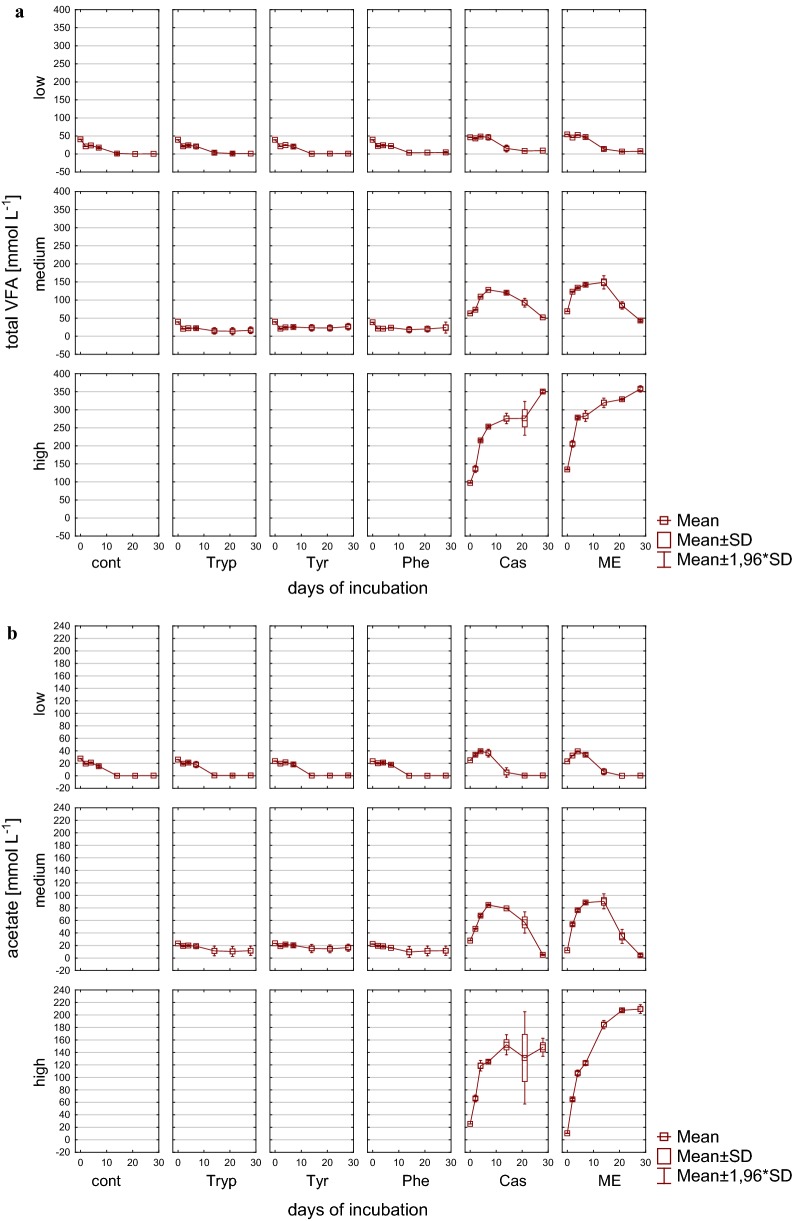
VFAs during 28 days of mesophilic anaerobic digestion from reactors reflecting different overload conditions (low, medium, high): **a** Sum of VFA (C1–C5) [mM]. **b** Acetate [mM]. *Cont* control, *Tryp* tryptophan, *Tyr* tyrosine, *Phe* phenylalanine, *ME* meat extract, *Cas* casein

**Fig. 4:**
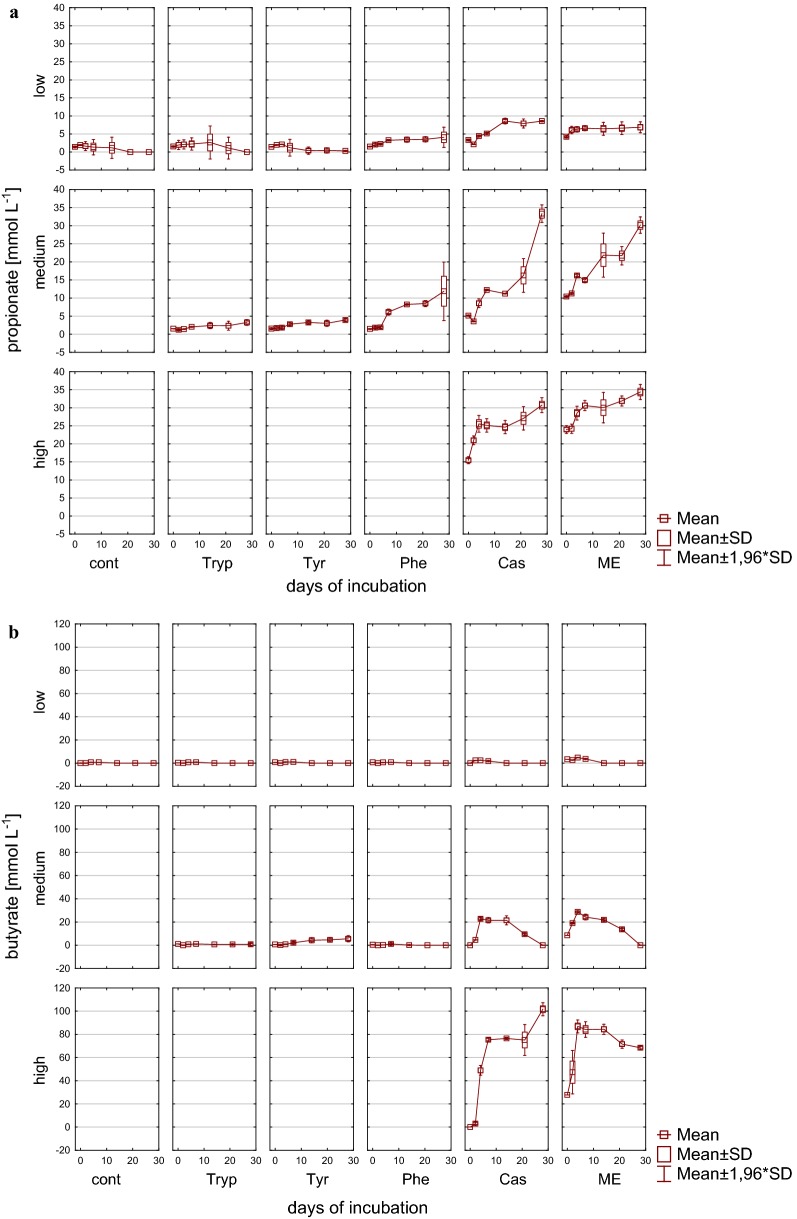
VFAs during 28 days of mesophilic anaerobic digestion from reactors reflecting different overload conditions (low, medium, high): **a** Propionate [mM]. **b** Butyrate [mM]. *Cont* control, *Tryp* tryptophan, *Tyr* tyrosine, *Phe* phenylalanine, *ME* meat extract, *Cas* casein

Accordingly, also VFA concentrations reflected the reactor overload conditions (Figs. 3, 4), particularly in reactors fed with complex substrates, whereas the alcohols methanol and ethanol could not be detected in concentrations exceeding 0.1 g L^−1^. The sum of VFA in these reactors showed an extremely strong increase within the first days of incubation under medium and high load conditions and exhibited an accumulation without any further degradation in high load reactors with up to 357.9 mM (± 4.30) C1–C5 VFA at the end of the incubation period. In contrast to high load reactors, the accumulation reversed with low and medium load (for complex substrates only after 14 days of incubation) and the microbial community from then on was able to convert butyrate into acetate and further into methane (Figs. 3b, 4b). In amino acid fed reactors, an accumulation of VFA was not noticeable but rather a decrease in the overall VFA pool which was mainly composed of acetate. Most likely acetate was used up by acetoclastic methanogenesis; however, in medium load amino acid fed reactors propionate tended to accumulate especially when phenylalanine was added as substrate (Fig. 4a). The effect of propionate accumulation (> 5 mM propionate) became even clearer in reactors fed with complex substrates. Therefore, propionate was not further degraded, even in reactors where total VFAs were decreasing and—particularly interesting—also even when butyrate was used up (Fig. 4b). Propionate degradation is thermodynamically an unfavorable process but coupled to syntrophic H_2_ utilization it becomes, similar to syntrophic butyrate oxidation, an exergonic process when the H_2_ partial pressure can be kept low by hydrogenotrophic methanogens [65]. The observed accumulation might, therefore, indicate an inhibited syntrophic propionate oxidation (see also below).

**Fig. 5:**
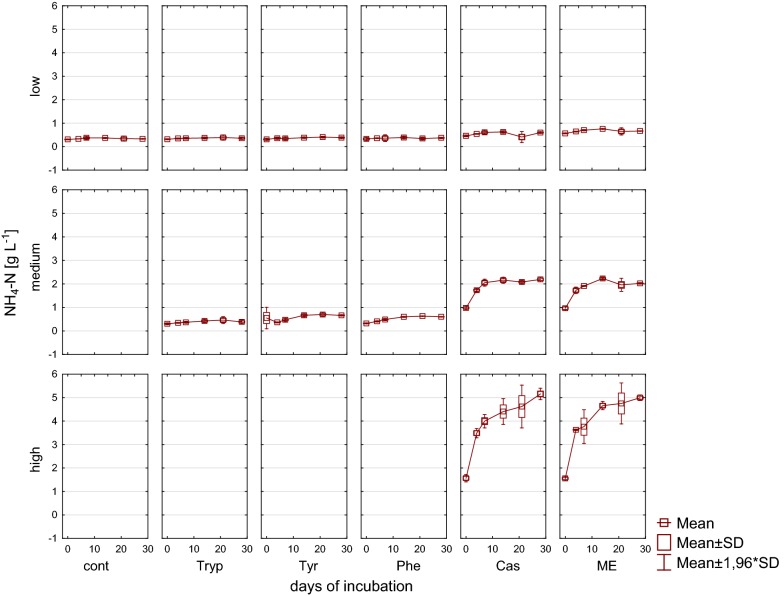
Ammonia nitrogen [g L^−1^] during 28 days of mesophilic incubation from reactors reflecting different overload conditions (low, medium, high). *Cont* control, *Tryp* tryptophan, *Tyr* tyrosine, *Phe* phenylalanine, *ME* meat extract, *Cas* casein

In reactors fed with complex substrates, an accumulation of NH_4_^+^ under medium and high load conditions was found when applying complex substrates (Fig. 5). For mesophilic digestions 3–5 g L^−1^ total ammonia concentration is thought to be manageable by an adopted microbial and methanogenic community [66]; at higher concentrations as observed in the present study for complex substrates under high load conditions, an inhibition by ammonia seems likely [67].

#### Formation of phenyl acids

**Fig. 6:**
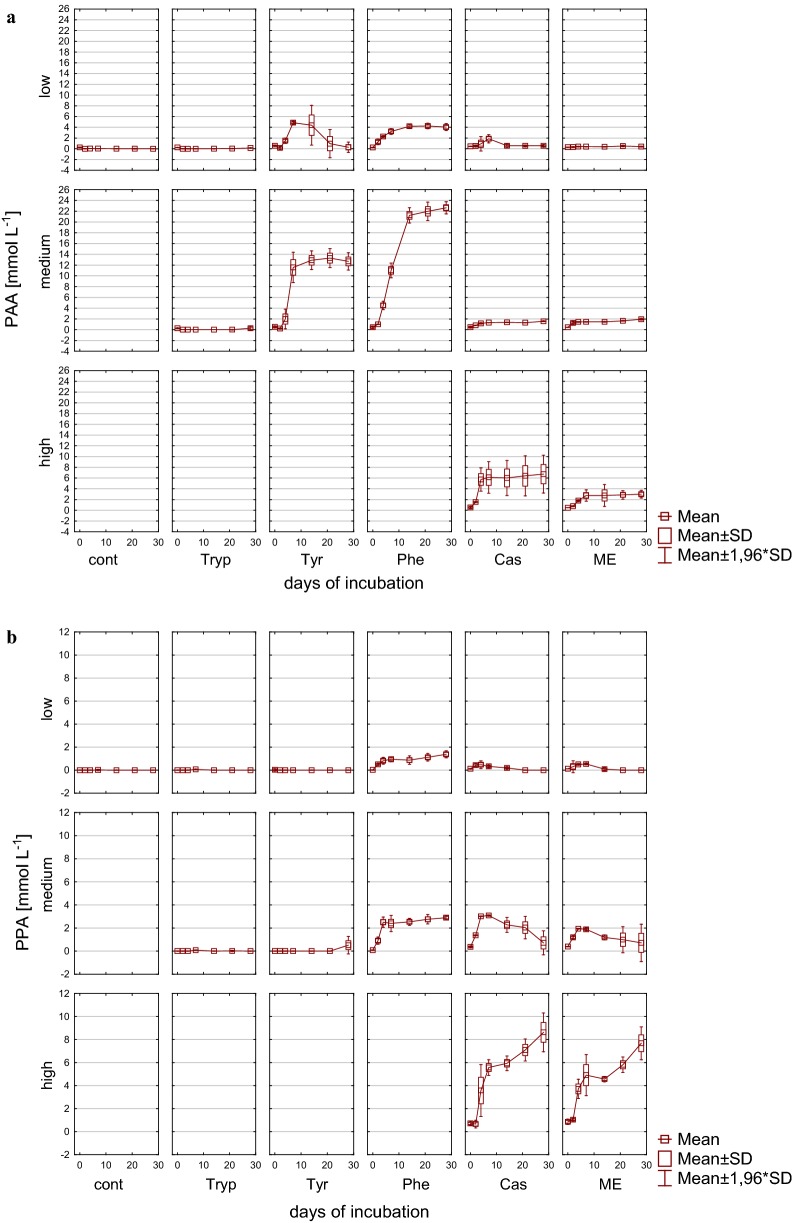
Formation of phenylacetic acid (PAA) (**a**) and phenylpropionic acid (PPA) (**b**) during mesophilic incubation from reactors reflecting different overload conditions (low, medium, high). *Cont* control, *Tryp* tryptophan, *Tyr* tyrosine, *Phe* phenylalanine, *ME* meat extract, *Cas* casein

With the exception of the controls, phenyl acids were formed during mesophilic incubation (Fig. 6) verifying that these acids are degradation products of precursor substances like the ones used throughout this investigation. The highest concentrations of phenyl acids could be determined for PAA in phenylalanine containing reactors, with an accumulation of 22.6 mM (± 0.58) PAA (~ 3070 mg L^−1^) followed by tyrosine with 12.7 mM (± 0.82) PAA(~1730 mg L^−1^) after 28 days of incubation. PAA is a direct degradation product of microbial phenylalanine decomposition derived from channeling reactions involved in the transformation to benzoyl-CoA [17], where it can be further degraded by an initial reduction of the aromatic ring followed by ring hydrolysis [18, 68, 69]. Concentrations previously shown to inhibit methanogenic activity were dependent on the substrate and increased with loading rates [4], and threshold concentrations ranged from 143 mg L^−1^ [5] to 3000 mg L^−1^ [7]. With up to 8.6 mM (± 0.86), the highest concentrations for PPA were found in reactors with complex protein-rich substrates with high load. Similar to PAA, also PPA is channeled to benzoyl-CoA where it is further degraded [17]. Generally, PPA concentrations were considerably lower than those for PAA. Therefore, amino acids tended to rather result in PAA formation, whereas complex, protein-rich substrates promoted the formation of PPA. In medium and high load reactors, phenyl acids accumulated and could not further be degraded until the end of the incubation period. Under low load conditions, by contrast, 4.9 mM (± 0.15) PAA which was formed until day 7 was almost entirely metabolized in tyrosine reactors until day 28 (Fig. 6a). PPA was also found to be degraded after its formation in complex protein-rich substrates under medium load conditions with both casein and meat extract as additional substrates.

Thermodynamic calculations indicated that the degradation of PAA under standard conditions (Table 3, reaction 1) was an endergonic process, whereas under the given mesophilic temperature regime and the applied settings it became exergonic in low load reactors within the first days of mesophilic incubation, in which the acetate pool was used up after 14 days (Figs. 3, 4), whereby a minimum of − 20 kJ mol^−1^ is considered necessary to make a microbial reaction thermodynamically feasible [70]. By contrast, in high load reactors acetate accumulated and ∆G′ values indicated unfavorable conditions for PAA degradation, which led to the overserved accumulation of PAA. In medium load reactors, however, from a thermodynamic point of view PAA degradation was feasible. Therefore, the found accumulation of phenyl acids was attributed to a faster generation from direct precursors like phenylalanine than their degradation was possible, all the more as the generation of PAA as a breakdown product of PPA following reaction 2 (Table 3) from a thermodynamic point of view was not possible.

Significant correlations (Spearman *p *< 0.01) of PAA were found with total carbon (*R*_Sp_ = 0.412), total nitrogen (*R*_Sp_ = 0.318), and NH_4_-nitrogen (*R*_Sp_ = 0.452) as well as of PPA with acetate (*R*_Sp_ = 0.568), butyrate (*R*_Sp_ = 0.567), sum of VFA (*R*_Sp_ = 0.662), total carbon (*R*_Sp_ = 0.726), total nitrogen (*R*_Sp_ = 0.686), NH_4_-nitrogen (*R*_Sp_ = 0.705), and C/N ratio (*R*_Sp_ = 0.705). Although higher concentrations of PAA (mean of 22.6 mM (± 0.58), ~3.07 g PAA L^−1^) were formed in total during the incubation time, correlations with PPA were generally stronger. A clear relationship of PAA and PPA generation and overload conditions could be confirmed.

In addition, a negative correlation with methane production could be observed when applying mesophilic incubation temperature. Considering the overall methane production as well as the methane production per carbon load, a negative impact of phenyl acids (sum) could be found (*R*_Sp_ = − 0.439 and *R*_Sp_ = − 0.622, respectively). Previous studies applying kitchen waste [4] or sugar beet pulp [7] as a substrate did not find a direct negative impact of PAA and PPA on methanogenic microorganisms in this study; the appearance of PAA and/or PPA per se did not mandatory result in a reduced methane generation. By contrast, Cabrol et al. [6] found an effect of PAA pulses of 200 mg L^−1^ on the microbial community structure of a primary sludge digester, which changed from an acetoclastic towards a hydrogenotrophic dominated one, whereas the biomass was resistant to repeated pulses of 600 mg L^−1^ in a mixed sludge digester. However, this needs further clarification by direct inhibition studies using the applied microbial communities or even pure cultures.

Particularly interesting are correlations of PAA and PPA with propionate (*R*_Sp_ = 0.530 and *R*_Sp_ = 0.754, respectively). As shown above, propionate degradation, which is mainly occurring syntrophically via the methyl-malonyl pathway in methanogenic habitats with succinic acid as symmetrical intermediate [71], was inhibited and the observed correlations suggest a possible link of phenyl acid and propionate accumulation. Taking tyrosine low load reactors as an example, propionate was found within the first days of incubation along with increasing PAA concentrations, whereas after 14 days PAA concentrations decreased and propionate was fully degraded. In contrast in tyrosine medium load reactors PPA accumulated and propionate was not further degraded. Therefore, a link seems possible; however, this has to be proven in further experiments.

### Thermophilic conditions

#### Reactor performance

As also observed for mesophilic conditions when applying thermophilic AD, methane production occurred in all tested samples exhibiting different stages of overload; however, to a varying extent dependent on the substrate used and the applied overload conditions. Final methane concentrations with thermophilic AD were higher than those observed under mesophilic conditions with up to 56% (± 1.5%) methane in control samples, whereas thermophilic medium and high load reactors resulted in higher end concentrations. In contrast, low load amino acid reactors reached a final methane concentration of 45–51%, while medium load conditions resulted in 30–48% methane. Hydrogen was detected in all samples during the first 4 days, but turned out to be highest in samples containing complex substrates and increased with substrate overload. Up to 12% (± 1.3%) H_2_ in high load, meat extract reactors were found at day 2 (please also refer to Additional file 1). Similar to mesophilic AD, the produced hydrogen was used up by the microbial community after 7 days of thermophilic incubation; however, medium and high load reactors did not entirely use up H_2_ and concentrations < 0.5% were still detectable.

**Fig. 7:**
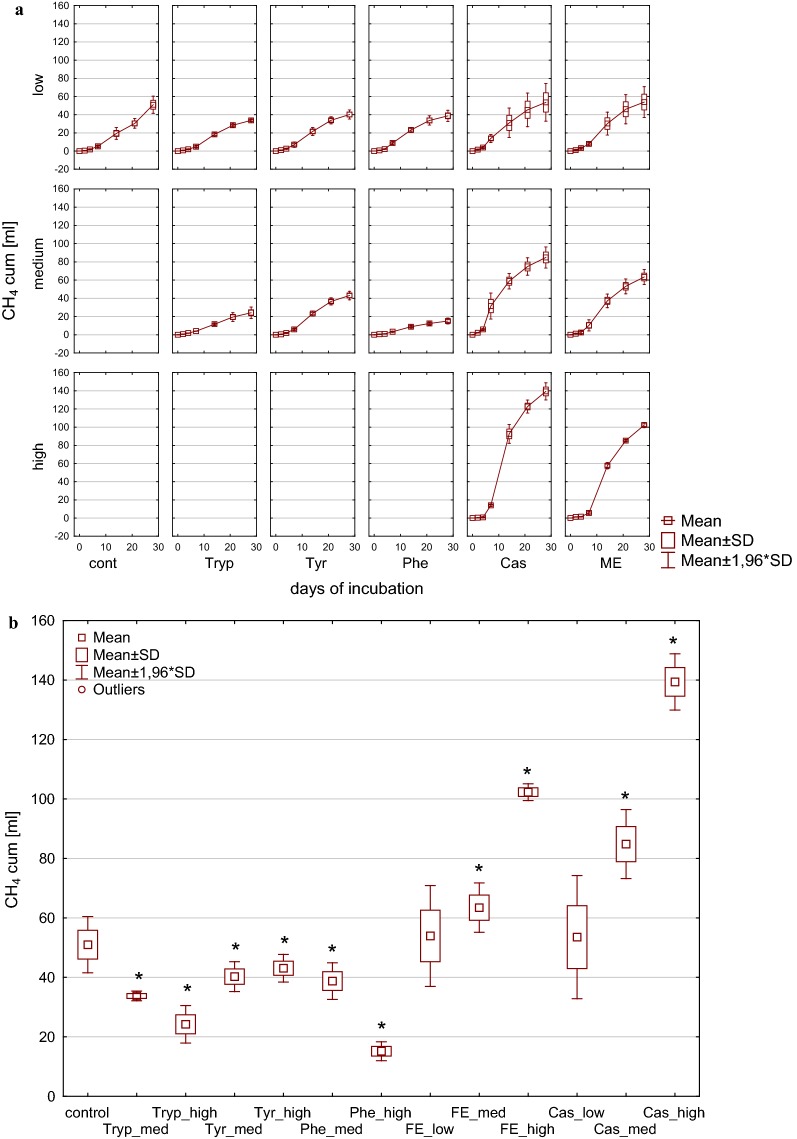
Cumulative methane production during (**a**) and total methane production (**b**) after 28 days of thermophilic incubation from reactors reflecting different overload conditions (low, medium, high). *Cont* control, *Tryp* tryptophan, *Tyr* tyrosine, *Phe* phenylalanine, *ME* meat extract, *Cas* casein. *Significantly different from control: conover test, *α* = 0.01, *H*_0_ rejected if *p *≤ *α*/2

Cumulative methane yield after 28 days of incubation as depicted in Fig. 7 revealed differences between the tested substrates. In all reactors, significant methane production commenced after a lag-phase of approx. 7 days. The application of amino acids as additional substrates led to significantly lower methane yields in comparison to controls (Fig. 7b). In contrast, meat extract and casein revealed significant differences regarding the different stages of overload with medium and high load reactors ending up with a significantly higher total methane production when compared to controls, whereas methane yield in low load reactors was not significantly different from that of controls. By comparing mesophilic and thermophilic incubation, the impact of overload under thermophilic conditions was less drastic resulting in the highest total methane production in reactors with the highest substrate concentrations.

**Fig. 8:**
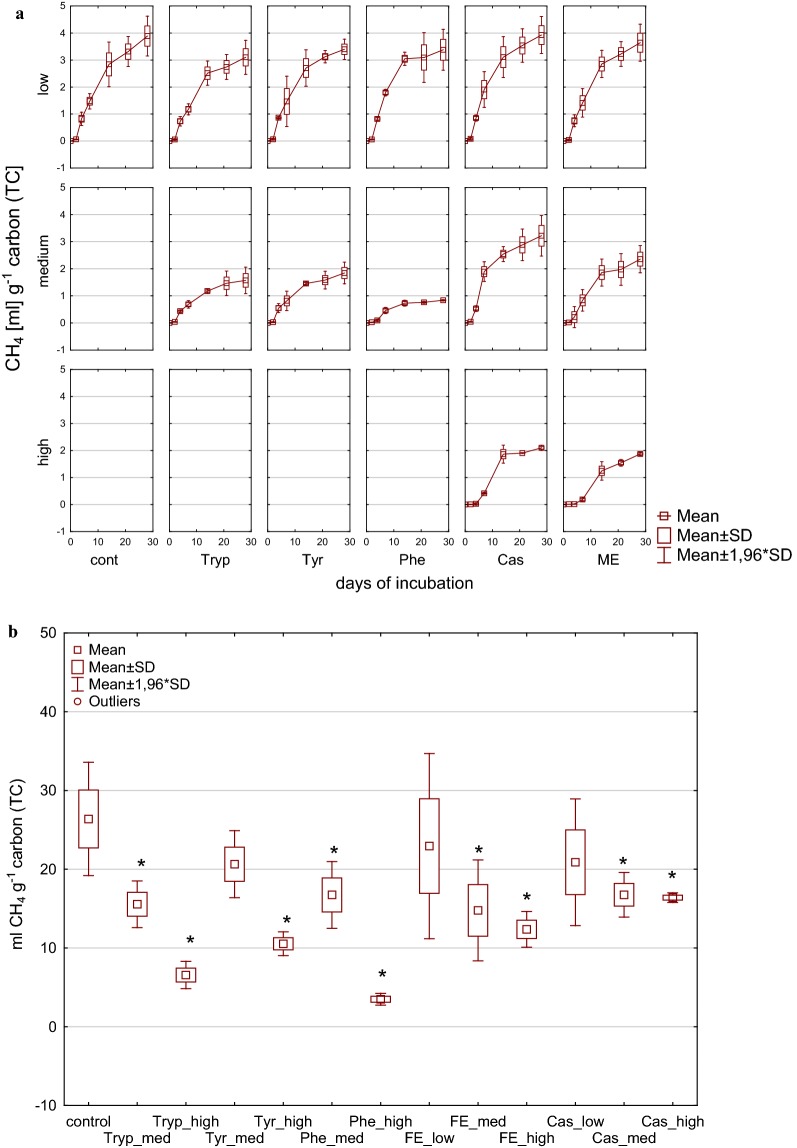
Methane yield per g carbon [mL CH_4_ g^−1^ TC] during (**a**) and at the end of (**b**) 28 days of thermophilic incubation from reactors reflecting different overload conditions (low, medium, high). *Cont* control, *Tryp* tryptophan, *Tyr* tyrosine, *Phe* phenylalanine, *ME* meat extract, *Cas* casein. *Significantly different from control: conover test, *α* = 0.01, *H*_0_ rejected if *p *≤ *α*/2

Considering the methane yield per carbon unit [mL CH_4_ g^−1^ TC] (Fig. 8) all amino acid fed reactors except tyrosine (low load) ended up with a lower methane yield compared with the controls as well as low load reactors produced significantly more methane than medium load ones. Therefore, an effect of an increased substrate pool (carbon and nitrogen) was obvious for these substrates. A similar influence of overload conditions was found for complex substrates, where low load reactors did not significantly reduce the methane yield per carbon compared to the control, whereas medium and high did. Therefore, the addition of complex substrates resulted in a significant difference of methane yield per carbon unit between low and medium, but not between medium and high load conditions.

**Fig. 9:**
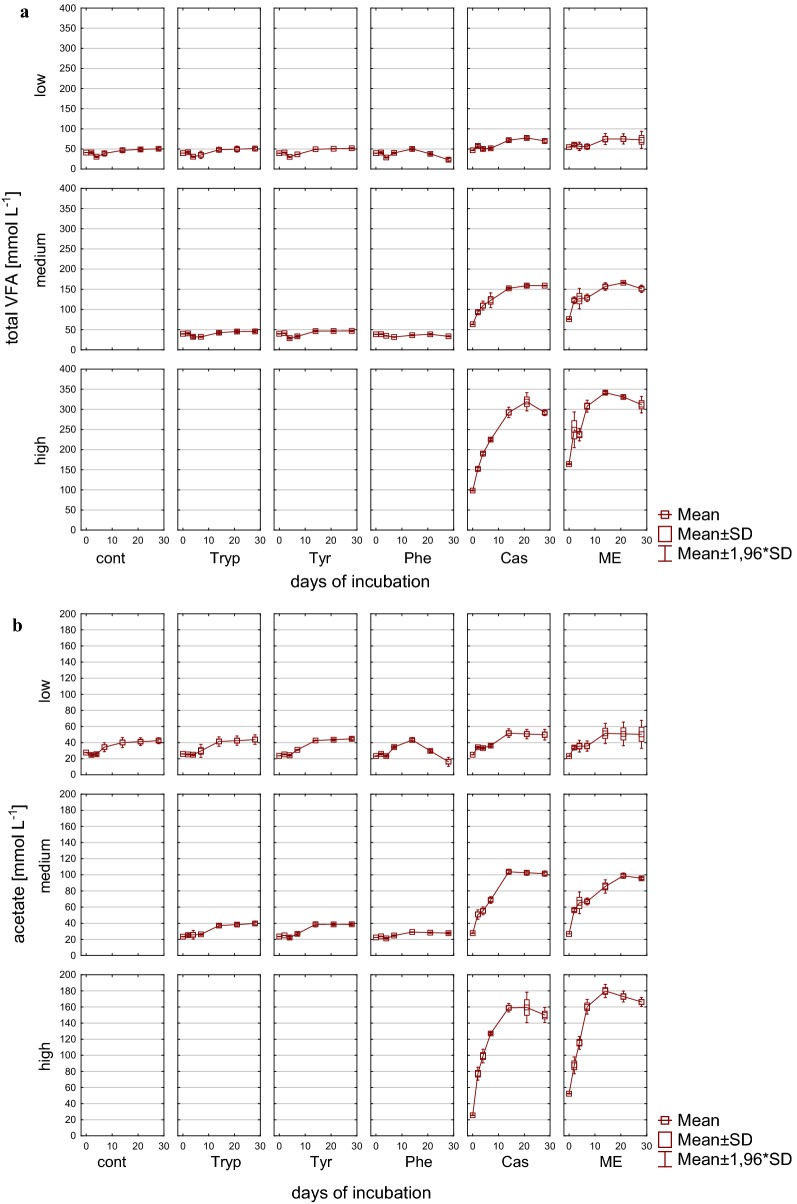
VFAs during 28 days of thermophilic anaerobic digestion from reactors reflecting different overload conditions (low, medium, high): **a** Sum of VFA (C1–C5) [mM]. **b** Acetate [mM]. *Cont* control, *Tryp* tryptophan, *Tyr* tyrosine, *Phe* phenylalanine, *ME* meat extract, *Cas* casein

**Fig. 10:**
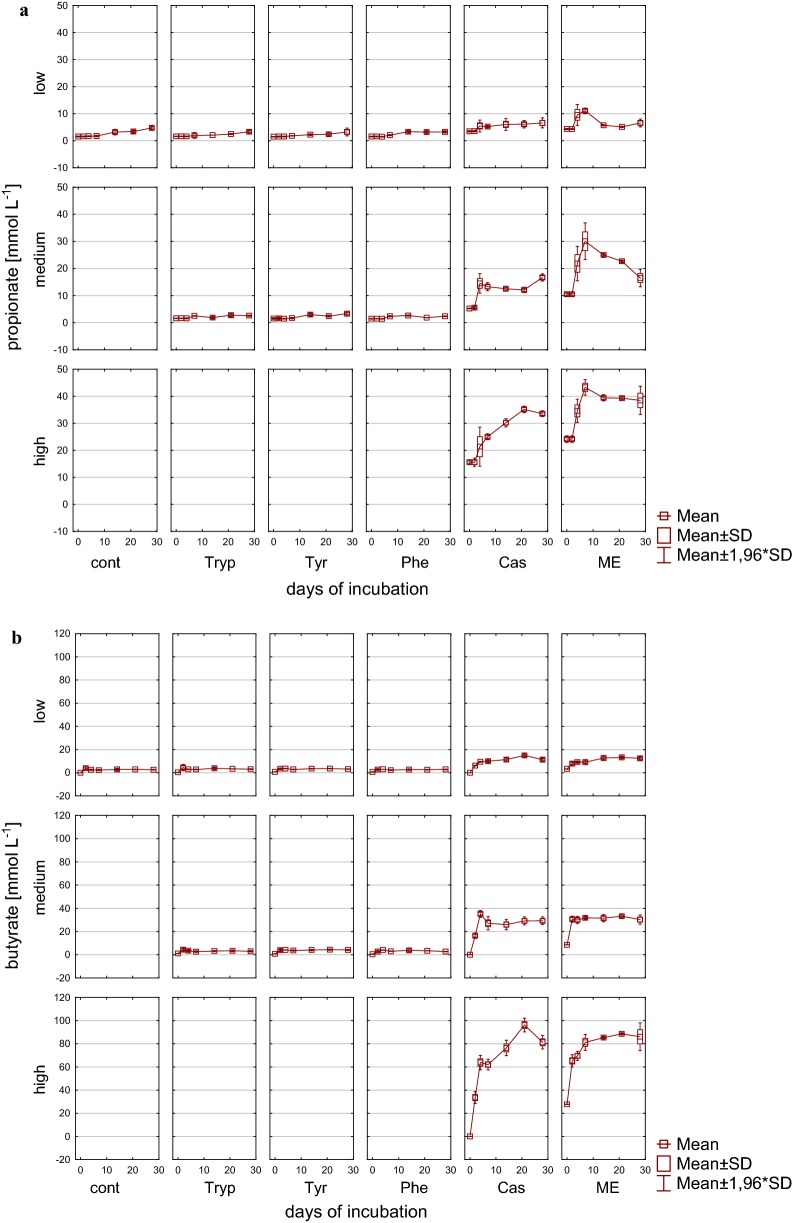
VFAs during 28 days of thermophilic anaerobic digestion  from reactors reflecting different overload conditions (low, medium, high): **a** Propionate [mM]. **b** Butyrate [mM]. *Cont* control, *Tryp* tryptophan, *Tyr* tyrosine, *Phe* phenylalanine, *ME* meat extract, *Cas* casein

VFA concentrations during the incubation period are depicted in Figs. 9 and 10. Similar to mesophilic AD methanol and ethanol could not be detected in concentrations exceeding 0.1 g L^−1^. Control reactors as well as amino acid fed ones showed similar total VFA concentrations that remained in the same range throughout the investigation period independently of the applied overload, while VFA concentrations in reactors fed with complex substrates increased during the incubation time. Low and medium complex substrate overload reactors tended to accumulate VFAs (Fig. 9a), whereas VFA concentrations reached a maximum under high load conditions at day 14 and 21, respectively, which then did not further increase or even started to decrease. The latter indicated a working microbial community that is adapted to very high substrate concentrations. While in controls and amino acid samples, the sum of VFAs was mainly composed of acetate, the VFA spectrum in complex substrates further included propionate and butyrate in high concentrations (Figs. 9, 10), both of which accumulated. In high load samples, a trend to acetate and butyrate degradation at the end of the incubation period was observed.

**Fig. 11:**
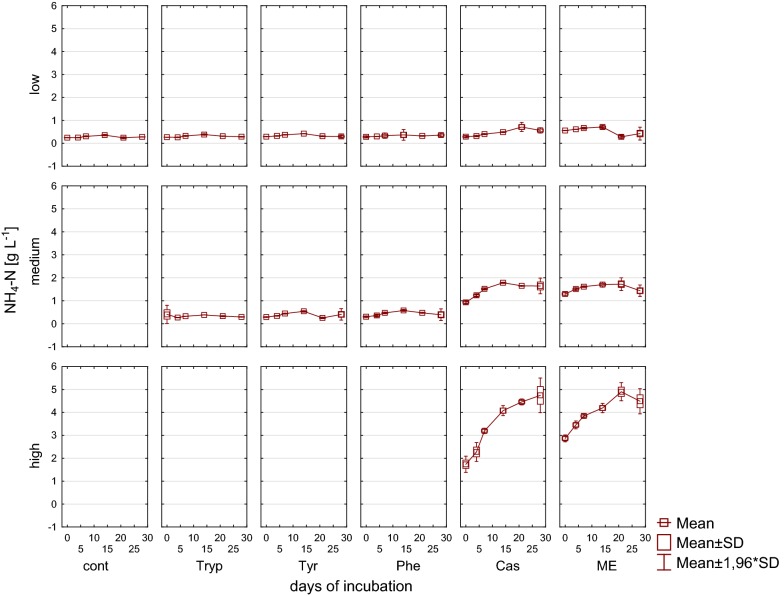
Ammonia nitrogen [g L^−1^] during 28 days of thermophilic incubation from reactors reflecting different overload conditions (low, medium, high). *Cont* control, *Tryp* tryptophan, *Tyr* tyrosine, *Phe* phenylalanine, *ME* meat extract, *Cas* casein

**Fig. 12:**
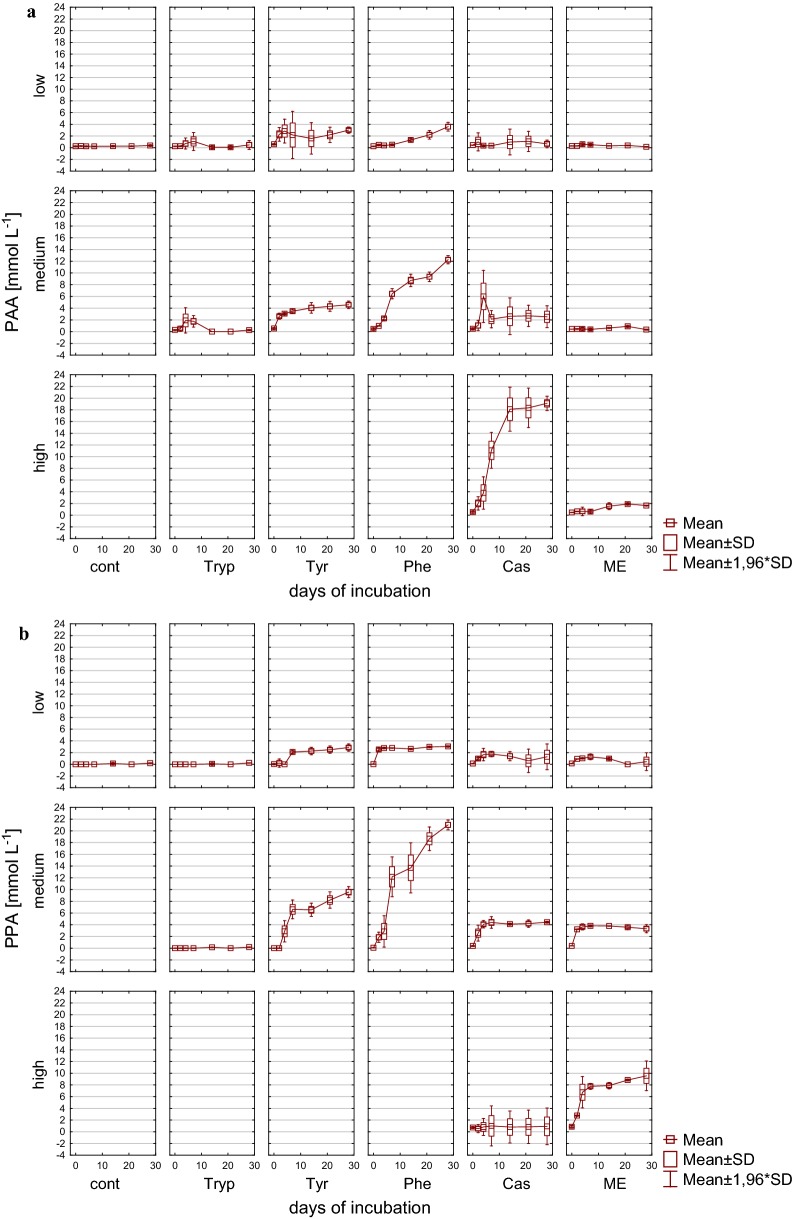
Formation of phenylacetic acid (PAA) (**a**), phenylpropionic acid (PPA) (**b**) during thermophilic incubation from reactors reflecting different overload conditions (low, medium, high). *Cont* control, *Tryp* tryptophan, *Tyr* tyrosine, *Phe* phenylalanine, *ME* meat extract, *Cas* casein

Furthermore, in reactors fed with complex substrates an accumulation of NH_4_^+^ was found; however, NH_4_^+^ concentrations stabilized or even tended to decrease at the end of the incubation period, indicating that the microbial community was able to handle these concentrations without a major inhibition (Fig. 11).

#### Formation of phenyl acids

Apart from the controls, phenyl acid formation was detected in all samples during thermophilic incubation (Fig. 12), with differences in the formation of PAA and PPA being obvious. While in amino acid fed reactors PAA accumulated with up to 12.3 mM (± 0.36) (phenylalanine, medium load), low concentrations of PAA were found in meat extract reactors irrespective of the applied overload condition. In casein fed reactors, in contrast, an overload-dependent increase in PAA concentration was found that resulted in concentrations up to 19.1 mM (± 0.62) PAA at the end of the incubation period. On the contrary, the highest PPA concentrations were found in phenylalanine and tyrosine fed reactors under medium load conditions [21.0 mM (± 0.43)], while the addition of meat extract led to concentrations of 9.6 mM (± 1.29) under high load conditions. Therefore, the presence of amino acids tended to result in PPA, whereas complex, protein-rich substrates promoted the accumulation of PAA under overload conditions in thermophilic AD.

By thermodynamic calculations, the degradation of PAA under standard conditions (Table 3, reaction 1) was shown to be an endergonic process, whereas under the given experimental setting it became exergonic in low and partly also in medium, but not in high load reactors. In contrast to mesophilic incubation, ∆G′ values, however, were near the energy limit of -20 kJ mol^−1^ necessary to make a microbial reaction feasible [70], since the acetate during thermophilic incubation was not completely used by the microbial community (Figs. 9, 10). The breakdown of PPA (Table 3, reaction 2 and 3) was unfavorable under both standard and the actual experimental conditions and became exergonic in only a few samples (e.g., casein medium load, Additional file 1: Figure S7).

Significant correlations (Spearman. *p *< 0.01) of PAA and PPA were found with total carbon (TC) (*R*_Sp_ = 0.232 and *R*_Sp_ = 0.284), total nitrogen (TN) (*R*_Sp_ = 0.336 and *R*_Sp_ = 0.310). NH_4_–N (*R*_Sp_ = 0.432 and *R*_Sp_ = 0.350), and butyrate (*R*_Sp_ = 0.376 and *R*_Sp_ = 0.307) as well as of PAA with acetate (*R*_Sp_ = 0.350) and propionate (*R*_Sp_ = 0.381). A relationship of PAA and PPA generation and overload conditions seems also likely for AD under thermophilic conditions; however, a correlation with total biogas or methane production could not be observed.

### Handling of overload under mesophilic and thermophilic conditions

**Fig. 13:**
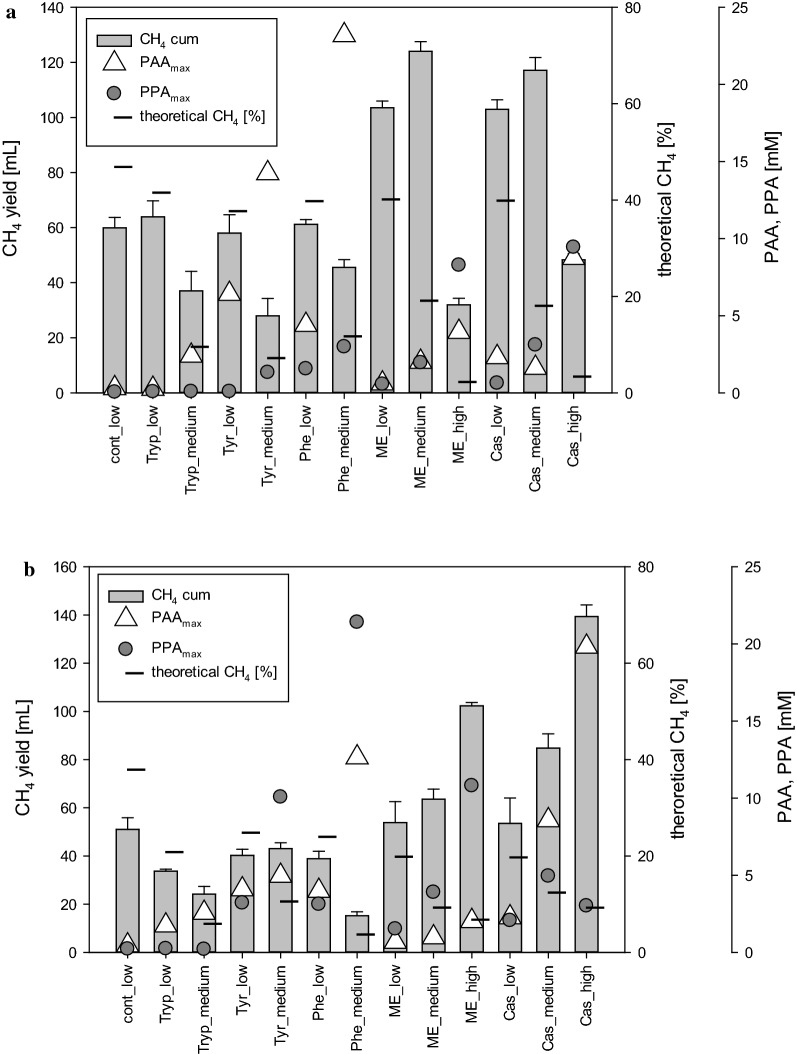
Methane yield, theoretical CH_4_ [%] and maximum concentrations of PAA and PPA during mesophilic (**a**) and thermophilic (**b**) incubation from reactors reflecting different overload conditions (low, medium, high). *Cont* control, *Tryp* tryptophan, *Tyr* tyrosine, *Phe* phenylalanine, *ME* meat extract, *Cas* casein

Control samples (without overload) showed similar methane concentrations and yields after 28 days of incubation irrespective of the applied temperature conditions. However, when the reactors were gradually overloaded, differences emerged based on the different overload levels under varied temperatures. During mesophilic incubation, reactors with low and medium load levels showed the highest methane production, whereas high complex substrate overload resulted in the highest methane generation during thermophilic AD; however, when compared with mesophilic conditions, with a prolonged lag phase at the beginning of the incubation. These differences might be explained by the different origins of the inocula, as the mesophilic one was derived from a wastewater treatment plant running under low load conditions [52], whereas the thermophilic one came from a solid state AD working with high loading rates [50]. The applied inocula not only influenced the ability of the microbial community to overcome unfavorable reactor conditions but also the potential occurrence of phenyl acids (Fig. 13).

As a result of substrate overloading in reactors fed with complex substrates, VFA and NH_4_^+^-concentrations increased and tended to accumulate under mesophilic conditions in high load reactors, while during thermophilic incubation these adverse effects were not apparent that clearly, as could be seen by decreasing acetate, propionate, and butyrate (Figs. 3, 4, 9, 10) as well as NH_4_^+^ concentrations at the end of the incubation period. However, this effect seems rather inoculum than temperature driven. In contrast, in the mesophilic reactors fed with complex substrates, an accumulation of VFAs and NH_4_^+^ was not found under low load conditions reflecting a working microbial degradation cascade. With an NH_4_-N concentration of more than 5 g L^−1^ in high load reactors, an inhibition of the microbial community not being adapted to such high concentrations seems likely [67, 72, 73].

The formation of phenyl acids is considered to occur before parameters like VFA or NH_4_^+^ concentrations, general indicators for process instability, would suggest overload conditions [4]. By the low detection limit of phenyl acids via HPLC analysis, this might allow addressing arising instabilities due to overload conditions earlier than traditional parameters like propionate would do [5]. However, additional knowledge on the microbial response to phenyl acids and on inhibition threshold concentrations for different substrates, operational circumstances, and the applied microbial communities is needed.

In the present investigation, the formation of phenyl acids, if taking place, was accompanied with an increase of VFA and NH_4_^+^ concentrations when applying complex substrates, whereas for amino acid fed reactors these effects were absent. Taking the overall process into account, correlations with parameters indicating reactor overload were found for mesophilic and, though in a lower extent, thermophilic conditions, thus pointing to a coherence of phenyl acid formation with overload conditions. During mesophilic incubation, a link of PAA and PPA generation and propionate accumulation was found pointing to an imbalanced syntrophic microbial community structure.

Although a negative correlation of phenyl acids with overall biogas or methane production was found under mesophilic conditions (but not for thermophilic), a negative impact of PAA and/or PPA on methanogenic archaea themselves seems not plausible. This is evidenced by the fact that the appearance of PAA or PPA did not mandatorily result in a decreased methane generation, although during mesophilic digestion high phenyl acid concentrations tended to result in reduced reactor performance (Fig. 6). Therefore, the formation of phenyl acids seems to adversely affect the microbial community downstream to the methanogenesis phase, even though Sierra-Alvarez and Lettinga [40] found an inhibitory effect of PAA on acetoclastic methanogenesis at PAA concentrations of 5.27 mM. The findings of the present study are in accordance with the findings of Hecht and Griehl [4], who investigated overload conditions with mixed kitchen wastes as substrate and did not consider PAA as a direct inhibitor of methanogenesis. Hence, methane production efficiency from biogas reactors dealing with elevated concentrations of phenyl acids is most likely determined by the microbial community structure, an effect that was also found previously [4, 42].

Phenylalanine was the substrate resulting in the highest phenyl acid concentrations in both mesophilic and thermophilic reactors, whereas tryptophan addition, another aromatic amino acid, resulted in minor concentrations during thermophilic AD and no formation during mesophilic incubation. Therefore, irrespective of the incubation temperature and the origin of the microbial community, phenylalanine seems to be one of the most important precursors of phenyl acids, all the more as PPA was previously described as a degradation product of phenylalanine metabolism [74, 75]. Phenylalanine as microbial degradation product in anaerobic digestion systems can derive from various proteinaceous substrates as well as lignocellulose containing resources [36]. Tyrosine, in contrast, a known precursor of PAA [75] in the present study led to the formation of PAA and PPA during thermophilic incubation, but solely to PAA formation in mesophilic AD.

Once formed, phenyl acids could also be catabolized, e.g., during thermophilic incubation of casein in medium load reactors (PAA) or in mesophilic with tyrosine under low load (PAA) and meat extract and casein also under medium load (PPA). As syntrophic interactions are thought to be required for phenyl acid degradation [10, 36, 76], with methanogenesis representing the most important final electron accepting reaction in AD systems [76], the methanogenic community remained intact during increased concentrations of phenyl acids and resulted in decreasing PAA and/or PPA concentrations later on. A previous investigation [26] showed that apart from benzoic acid, none of various other tested aromatic compounds was mineralized by the thermophilic community incubated at 55 °C, suggesting that channeling reactions to the central intermediate benzoyl-CoA were inoperative in this microbial community. However, in their investigation, decrease in the temperature below 50 °C triggered the degradation of phenols, most probably caused by a negative effect on enzyme activities [26]. In general, phenol-degrading microorganisms have been isolated mainly from mesophilic habitats [21, 26, 77, 78]; however, only a few studies have isolated the impact of temperature on the generation of phenyl acids. In the present investigation, a vague trend of better degradability of phenyl acids via mesophilic conditions could be found; however, further investigation are necessary to better understand the dynamics of phenyl acid formation, accumulation, and degradation by the applied microbial consortia under different temperature regimes.

## Conclusions

Summarizing the findings of this study, it can be noted that

1. The applied substrates led to the formation of phenyl acids PAA and PPA.
2. The effect of phenyl acid formation was mainly substrate load dependent.
3. The formation of phenyl acids was less inoculum and/or temperature than substrate driven; which of the two phenyl acids was predominantly produced was temperature/inoculum driven.
4. Once formed, the formation of phenyl acids constitutes a reversible process during mesophilic AD, while during thermophilic incubation phenyl acids tended to accumulate without further degradation.
5. PAA and PPA might be interesting intermediates for process monitoring due to their correlation with reactor overload conditions and other parameters indicating community imbalances (e.g., syntrophic propionate oxidation) in combination with their high UV absorption and, therefore, low detection limit via HPLC analysis.

The hypothesis that phenyl acids formed during overload conditions in anaerobic digestion reactors would generally inhibit the methanation process had to be rejected. However, phenyl acids seem to play an important role in the microbial response to overloaded biogas systems and need further investigation to gain a better understanding of their role as well as the microbial interactions leading to the formation of those acids.

## Additional file

**Additional file 1: Figure S1.** Hydrogen concentration in the headspace [%] during 28 days of mesophilic incubation from reactors reflecting different overload conditions (low, medium, high). *Cont* control, *Tryp* tryptophan, *Tyr* tyrosine, *Phe* phenylalanine, *ME* meat extract, *Cas* casein. **Figure S2.** Hydrogen concentration in the headspace [%] during 28 days of thermophilic incubation from reactors reflecting different overload conditions (low, medium, high). *Cont* control, *Tryp* tryptophan, *Tyr* tyrosine, *Phe* phenylalanine, *ME* meat extract, *Cas* casein. **Figure S3.** pH measured via indicator strips (Dosatest, VWR, Germany) during 28 days of mesophilic incubation from reactors reflecting different overload conditions (low, medium, high). *Cont* control, *Tryp* tryptophan, *Tyr* tyrosine, *Phe* phenylalanine, *ME* meat extract, *Cas* casein. **Figure S4.** pH measured via indicator strips (Dosatest, VWR, Germany) during 28 days of thermophilic incubation from reactors reflecting different overload conditions (low, medium, high). *Cont* control, *Tryp* tryptophan, *Tyr* tyrosine, *Phe* phenylalanine, *ME* meat extract, *Cas* casein. **Figure S5.** NH_3_ concentration during 28 days of mesophilic incubation from reactors reflecting different overload conditions (low, medium, high). *Cont* control, *Tryp* tryptophan, *Tyr* tyrosine, *Phe* phenylalanine, *ME* meat extract, *Cas* casein. **Figure S6.** NH_3_ concentration during 28 days of thermophilic incubation from reactors reflecting different overload conditions (low, medium, high). *Cont* control, *Tryp* tryptophan, *Tyr* tyrosine, *Phe* phenylalanine, *ME* meat extract, *Cas* casein. **Figure S7.** PAA degradation during the first days of thermophilic incubation (A) and PPA accumulation (B) with ∆G′ values for reaction 1 (A) and reactions 2, 3 according to Table 3. A minimum of − 20 kJ mol^−1^ was considered necessary to make a microbial reaction thermodynamically feasible [70]. **Table S1.** Total carbon [g L^−1^], total nitrogen [g L^−1^], and C/N ratio after 28 days of mesophilic incubation from reactors reflecting different overload conditions (low, medium, high). *Cont* control, *Tryp* tryptophan, *Tyr* tyrosine, *Phe* phenylalanine, *ME* meat extract, *Cas* casein. **Table S2.** Total carbon [g L^−1^], total nitrogen [g L^−1^], and C/N ratio after 28 days of thermophilic incubation from reactors reflecting different overload conditions (low, medium, high). *Cont* control, *Tryp* tryptophan, *Tyr* tyrosine, *Phe* phenylalanine, *ME* meat extract, *Cas* casein

## Abbreviations

∆G′: Gibb’s free energy
∆G^0^′: standard Gibb’s free energy
∆G_f_^0^: standard free enthalpy of formation
AD: anaerobic digestion
C/N ratio: carbon:nitrogen ration
CMC: carboxymethylcellulose
CMCM: carboxymethylcellulose medium
COD: chemical oxygen demand
FW: fresh weight
GC: gaschromatography
HPLC: high-performance liquid chromatography
HPLC–RI: refractive index detector
HPLC–UV/VIS: UV/VIS detector
NAC: *N*-acetylcystein
NPOC: non-purgeable organic carbon
OPA: *ortho*-phthaldialdehyd
PAA: phenylacetate
PBA: phenylbutyrate
PPA: phenylpropionate
TC: total carbon
TN: total nitrogen
TS: total solids
VFA: volatile fatty acid
VS: volatile solids
