# Supplementary material for: Formation of phenylacetic acid and phenylpropionic acid under different overload conditions during mesophilic and thermophilic anaerobic digestion
Source: Biotechnol Biofuels. 2019 Feb 10;12:26. doi: 10.1186/s13068-019-1370-6 (PMC6368962; doi:10.1186/s13068-019-1370-6)
Supplement: Supplementary file 1 — Additional file 1: Figure S1. Hydrogen concentration in the headspace [%] during 28 days of mesophilic incubation from reactors reflecting different overload conditions (low, medium, high). Cont control, Tryp tryptophan, Tyr tyrosine, Phe phenylalanine, ME meat extract, Cas casein. Figure S2. Hydrogen concentration in the headspace [%] during 28 days of thermophilic incubation from reactors reflecting different overload conditions (low, medium, high). Cont control, Tryp tryptophan, Tyr tyrosine, Phe phenylalanine, ME meat extract, Cas casein. Figure S3. pH measured via indicator strips (Dosatest, VWR, Germany) during 28 days of mesophilic incubation from reactors reflecting different overload conditions (low, medium, high). Cont control, Tryp tryptophan, Tyr tyrosine, Phe phenylalanine, ME meat extract, Cas casein. Figure S4. pH measured via indicator strips (Dosatest, VWR, Germany) during 28 days of thermophilic incubation from reactors reflecting different overload conditions (low, medium, high). Cont control, Tryp tryptophan, Tyr tyrosine, Phe phenylalanine, ME meat extract, Cas casein. Figure S5. NH3 concentration during 28 days of mesophilic incubation from reactors reflecting different overload conditions (low, medium, high). Cont control, Tryp tryptophan, Tyr tyrosine, Phe phenylalanine, ME meat extract, Cas casein. Figure S6. NH3 concentration during 28 days of thermophilic incubation from reactors reflecting different overload conditions (low, medium, high). Cont control, Tryp tryptophan, Tyr tyrosine, Phe phenylalanine, ME meat extract, Cas casein. Figure S7. PAA degradation during the first days of thermophilic incubation (A) and PPA accumulation (B) with ∆G′ values for reaction 1 (A) and reactions 2, 3 according to Table 3. A minimum of − 20 kJ mol−1 was considered necessary to make a microbial reaction thermodynamically feasible [70]. Table S1. Total carbon [g L−1], total nitrogen [g L−1], and C/N ratio after 28 days of mesophilic incubation from reacto [file 13068_2019_1370_MOESM1_ESM.docx]

Additional Figure S1. Hydrogen concentration in the headspace [%] during 28 days of mesophilic incubation from flasks reflecting different overload conditions (low, medium, high). Cont: control; Tryp: tryptophan; Tyr: tyrosine; Phe: phenylalanine; FE: meat extract; Cas: casein.

Additional Figure S2. Hydrogen concentration in the headspace [%] during 28 days of thermophilic incubation from flasks reflecting different overload conditions (low, medium, high). Cont: control; Tryp: tryptophan; Tyr: tyrosine; Phe: phenylalanine; FE: meat extract; Cas: casein.

Additional Figure S3. pH measured via indicator strips (Dosatest, VWR, Germany) during 28 days of mesophilic incubation from flasks reflecting different overload conditions (low, medium, high). Cont: control; Tryp: tryptophan; Tyr: tyrosine; Phe: phenylalanine; FE: meat extract; Cas: casein.

Additional Figure S4. pH measured via indicator strips (Dosatest, VWR, Germany) during 28 days of thermophilic incubation from flasks reflecting different overload conditions (low, medium, high). Cont: control; Tryp: tryptophan; Tyr: tyrosine; Phe: phenylalanine; FE: meat extract; Cas: casein.

Additional Figure S5. NH_3_ concentration during 28 days of mesophilic incubation from flasks reflecting different overload conditions (low, medium, high). Cont: control; Tryp: tryptophan; Tyr: tyrosine; Phe: phenylalanine; FE: meat extract; Cas: casein.

Additional Figure S6. NH_3_ concentration during 28 days of thermophilic incubation from flasks reflecting different overload conditions (low, medium, high). Cont: control; Tryp: tryptophan; Tyr: tyrosine; Phe: phenylalanine; FE: meat extract; Cas: casein.

**B**

**A**

Additional Figure S7. PAA degradation during the first days of thermophilic incubation (A) and PPA accumulation (B) with ∆G’ values for reaction 1 (A) and reactions 2, 3 according to Table 3. A minimum of -20 kJ mol^-1^ was considered necessary to make a microbial reaction thermodynamically feasible [70].

Additional Table S1. Total carbon [g L^-1^], total nitrogen [g L^-1^], and C/N ratio after 28 days of mesophilic incubation from flasks reflecting different overload conditions (low, medium, high). Cont: control; Tryp: tryptophan; Tyr: tyrosine; Phe: phenylalanine; FE: meat extract; Cas: casein.

| **substrate** | **overload** | **TC (+/- SD)**  **[g L^-1^]** | **TN (+/- SD)**  **[g L^-1^]** | **C/N ratio (+/- SD)** |
| --- | --- | --- | --- | --- |
| **control** |  | 2.8 (0.37) | 0.4 (0.01) | 7.7 (0.72) |
| **tryptophan** | low | 3.1 (0.43) | 0.4 (0.05) | 7.1 (0.52) |
| **tryptophan** | medium | 7.0 (0.25) | 1.0 (0.09) | 7.2 (0.42) |
| **tyrosine** | low | 3.5 (0.25) | 0.4 (0.02) | 8.3 (0.97) |
| **tyrosine** | medium | 5.5 (0.23) | 0.8 (0.09) | 6.9 (0.85) |
| **phenylalanine** | low | 3.3 (0.23) | 0.4 (0.02) | 8.4 (0.35) |
| **phenylalanine** | medium | 8.9 (0.10) | 1.0 (0.02) | 9.1 (0.16) |
| **meat extract** | low | 3.3 (0.56) | 0.8 (0.08) | 4.2 (0.34) |
| **meat extract** | medium | 6.4 (0.47) | 2.1 (0.09) | 3.1 (0.2) |
| **meat extract** | high | 17.7 (1.89) | 5.2 (0.08) | 3.4 (0.41) |
| **casein** | low | 3.0 (0.53) | 0.7 (0.10) | 4.4 (0.16) |
| **casein** | medium | 7.3 (0.10) | 2.2 (0.04) | 3.3 (0.1) |
| **casein** | high | 17.3 (2.37) | 5.3 (0.05) | 3.3 (0.47) |

Additional Table S2. Total carbon [g L^-1^], total nitrogen [g L^-1^], and C/N ratio after 28 days of thermophilic incubation from flasks reflecting different overload conditions (low, medium, high). Cont: control; Tryp: tryptophan; Tyr: tyrosine; Phe: phenylalanine; FE: meat extract; Cas: casein.

| **substrate** | **overload** | **TC (+/- SD)**  **[g L^-1^]** | **TN (+/- SD)**  **[g L^-1^]** | **C/N ratio (+/- SD)** |
| --- | --- | --- | --- | --- |
| **control** |  | 1.9 (0.08) | 0.4 (0.02) | 5.4 (0.23) |
| **tryptophan** | low | 2.2 (0.16) | 0.4 (0.01) | 5.3 (0.23) |
| **tryptophan** | medium | 3.7 (0.05) | 0.9 (0.02) | 4.1 (0.06) |
| **tyrosine** | low | 2.0 (0.19) | 0.3 (0.03) | 5.8 (0.04) |
| **tyrosine** | medium | 4.1 (0.21) | 0.8 (0.08) | 5.1 (0.28) |
| **phenylalanine** | low | 2.3 (0.27) | 0.4 (0.03) | 5.7 (0.23) |
| **phenylalanine** | medium | 4.3 (0.02) | 0.8 (0.01) | 5.1 (0.03) |
| **meat extract** | low | 2.4 (0.22) | 0.7 (0.05) | 3.5 (0.09) |
| **meat extract** | medium | 4.4 (0.69) | 1.9 (0.29) | 2.4 (0.02) |
| **meat extract** | high | 8.3 (0.73) | 4.6 (0.31) | 1.8 (0.24) |
| **casein** | low | 2.6 (0.25) | 0.7 (0.08) | 3.5 (0.01) |
| **casein** | medium | 5.1 (0.11) | 2.0 (0.20) | 2.6 (0.28) |
| **casein** | high | 8.5 (0.16) | 5.1 (0.63) | 1.7 (0.17) |
